# Exploration of Suitable Conditions for Shoot Proliferation and Rooting of *Quercus robur* L. in Plant Tissue Culture Technology

**DOI:** 10.3390/life15030348

**Published:** 2025-02-23

**Authors:** Ting Wang, Hao Li, Jiujiu Zhao, Jinliang Huang, Yu Zhong, Zhenfeng Xu, Fang He

**Affiliations:** National Forestry and Grassland Administration Key Laboratory of Forest Resources Conservation and Ecological Safety on the Upper Reaches of the Yangtze River & Forestry Ecological Engineering in the Upper Reaches of the Yangtze River Key Laboratory of Sichuan Province, College of Forestry, Sichuan Agricultural University, Chengdu 611130, China; wangting96@sicau.edu.cn (T.W.); 202001243@stu.sicau.edu.cn (H.L.); zhaojiujiu@stu.sicau.edu.cn (J.Z.); 80204@sicau.edu.cn (J.H.); zhongyu315@163.com (Y.Z.); xuzf@sicau.edu.cn (Z.X.)

**Keywords:** *Quercus robur* L., tissue culture, shoot proliferation, rooting

## Abstract

*Quercus robur* L., also referred to as “summer oak” or “English oak”, is an esthetically pleasing species, making it an excellent choice for street trees and gardens. Raising *Quercus* presents several challenges, including its long growth period, delayed germination, and inconsistent emergence. The shoot proliferation and adventitious root formation of *Q. robur* are crucial for establishing a tissue culture regeneration system and are vital for the successful transplantation of seedlings. To address this, experiments were conducted to assess shoot proliferation and adventitious root formation in *Q. robur* using various media. The shoot proliferation time, shoot proliferation coefficient, number of rooting strips, and length indicators of roots were recorded. The results indicated that a combination of 0.3 mg/L 6-Benzylaminopurine (6-BA) and 100 mg/L cefotaxime (Cef) was optimal for shoot propagation, while a solution of 0.1 mg/L 1-Naphthaleneacetic acid (NAA) and 1/2 Murashige and Skoog Medium (1/2MS) medium was most effective for root induction. This study has identified the optimal conditions for adventitious root formation and shoot proliferation in *Q. robur*, providing a basis for further research into propagation, germplasm conservation and genetic transformation techniques.

## 1. Introduction

*Quercus*, colloquially known as oak, refers to a group of plants within the genus *Quercus*, which belongs to the family Fagaceae. *Quercus* is extensively distributed, predominantly in the Northern Hemisphere, and is recognized for its significant ecological and economic value [1,2]. The timber of this species is robust, making it suitable for construction and furniture applications [3,4]. *Quercus robur* is considered a superior tree species due to its higher chlorophyll content and enhanced growth [5]. Additionally, *Q. robur* exhibits deep root systems and is resistant to drought, short-term flooding, strong winds, and air pollution [6]. Moreover, this species demonstrates greater tolerance to salinity and alkaline conditions, exhibiting a broader adaptability range, and can thrive under arid and infertile gravelly sandy loam soil conditions, playing a crucial role in soil and water conservation as well as air purification [7]. However, there are certain limitations in utilizing *Quercus* resources in production practice. *Quercus* contains tannic acid and other compounds that impede rooting, rendering it challenging to propagate via cuttings, with the literature on successful rooting outcomes being scant [8]. The propagation of *Quercus* is currently predominantly achieved through seed propagation, but seed propagation has several disadvantages, such as an extended growth cycle [9], delayed fruiting, reduced yields [10], and instability [11]. Furthermore, *Quercus* seeds are challenging to preserve over extended periods, with stringent storage conditions required [12]. Tissue culture breeding can compensate for the deficiencies inherent in traditional breeding methods. Moreover, optimized tissue culture techniques are the basis for efforts to conserve plant genetic resources and breeding [13,14]. Consequently, the shoot proliferation culture and adventitious root formation of *Q. robur* are pivotal components in the development of a tissue culture regeneration system for this species.

At present, plant tissue culture is widely applied in the propagation of oak species, with successful outcomes reported in a few select oak varieties [15]. However, challenges remain, including the low rooting and survival rates of tissue culture for some *Quercus* species, necessitating further experimentation. Exogenous hormones play a crucial role in plant tissue culture [16]. Cytokinins, including thidiazuron (TDZ), 6-benzyl aminopurine (6-BA), and kinetin (KT), are primarily utilized to stimulate cell division and differentiation, thus facilitating shoot regeneration [17,18,19]. The specific types and concentrations of these hormones significantly influence the morphology and regeneration rate of explants [20]. Antibiotics are frequently employed to prevent contamination of plant tissue culture and can suppress or even eradicate other microorganisms at low concentrations [21]. To balance the growth of endophytes in plants, antibiotics are usually added into the medium [22]. However, antibiotics may adversely affect shoot proliferation and differentiation [23]. Various species of explants exhibit diverse responses to varying antibiotic concentrations, with high concentrations potentially inhibiting shoot tip regeneration [24]. In addition, different medium substrates can have different effects on the plant tissue culture process. Consequently, it has been observed that the effectiveness of these hormones in promoting shoot proliferation and adventitious root formation in *Q. robur* is not yet stable. Thus, it is imperative to further investigate the technical system governing adventitious shoot proliferation and root formation in *Q. robur*.

Shoot proliferation and adventitious root formation are critical for the post-transplantation survival of seedlings, underscoring the importance of optimizing medium formulations to enhance these processes in *Q. robur*. By integrating various exogenous hormones, antibiotics, and types of culture medium, we have derived an improved formulation for the proliferation of *Q. robur* shoots and the induction of adventitious root formation. The novelty of our study lies in exploring the differences between different antibiotics and different media, which proved to be highly significant for shoot proliferation and adventitious root formation in *Q. robur*. Furthermore, our experimental results demonstrated significantly higher efficiency in both rooting and shoot proliferation compared to previous studies, making our findings particularly noteworthy. This provides robust technical support for the rapid propagation of *Q. robur* and lays a theoretical foundation for the utilization of its germplasm resources and the realization of its ecological benefits.

## 2. Materials and Methods

### 2.1. Obtaining Sterile Materials of Q. robur

The materials used in this study were seeds sourced from 20-year-old *Q. robur* trees, collected in Finland (60°1′ N, 24°53′ E) in October 2021. The experiment was carried out as soon as we received the seeds, while leaving a portion of the seeds in a dry room temperature storage cabinet. Seeds were initially dehusked to extract the zygotic embryos. Following this, the zygotic embryos were subjected to continuous water rinsing for 4 h and then immersed in a 75% ethanol solution for 1.5 min. The embryos were rinsed once with sterile water to remove any residual ethanol. The embryos were then disinfected by immersing them in a 5% sodium hypochlorite solution with active chloride for a duration of 10 min. After disinfection, the embryos were rinsed three times with sterile water to ensure the complete removal of the disinfectant. After the previous protocol of sterilization, these successfully sanitized seeds were then cultured in a growth medium to induce germination and root development, ultimately leading to the cultivation of oak tree seedlings. The composition of the growth medium used was as follows: Woody Plant Medium (WPM) supplemented with 30 g/L sucrose and 6 g/L agar, but no phytohormones were added, with the pH between 5.70 and 5.84 [25]. All the seeds and subsequent seedlings were cultivated in a growth chamber at Sichuan Agricultural University in Chengdu, Sichuan. The temperature was maintained at 25 °C, with a photoperiod of 16 h and a photosynthetic active radiation (PAR) intensity of 140 µmol m^−2^ s^−1^. The relative humidity was kept at 60%.

We used 20 seeds for germination and then selected the most robustly germinated ones for subsequent experiments to maintain the genetic uniformity and stability of the experimental material. In order to obtain a large number of seedlings, the selected seedlings were propagated on a medium, and the formulation of it was WPM supplemented with 30 g/L sucrose, 6 g/L agar, and 0.6 mg/L N-(phenylmethyl)-9-(tetrahydro-2H-pyran-2-yl)-9H-purin-6-amine (PBA), with the pH between 5.70 and 5.84 [25]. This medium was also suitable for the subsequent subculture, and a substantial amount of propagation material was harvested from proliferation cultures of *Q. robur* via constant successive subcultures once a month.

### 2.2. Determination of Optimal Phytohormone Concentration for Adventitious Shoot Proliferation

From the 8th subculture, adventitious shoots were selected for experimentation 30 days post cultivation, with the length of the shoots approximately 3 cm and at least one leaf. Using WPM as the basal culture medium, supplemented with 30 g/L of sucrose, 6 g/L of agar, and 75 mg/L of the antibiotic cefotaxime (Cef), the pH was adjusted to between 5.70 and 5.84. Different concentrations of TDZ (BIORIGIN, 82BN20275) (0.002, 0.004, 0.01, 0.02, and 0.05 mg/L) or 6-BA (BIORIGIN, BN20251) (0.1, 0.2, 0.3, 0.4, and 0.5 mg/L) were added to the basal medium to promote the proliferation of the shoots (Supplemental Table S1). Each treatment was prepared with 15 shoots twice, equivalent to 30 replicates. The number of days to achieve 80% shoot proliferation was recorded. The number of proliferated shoots was counted after 30 days of inoculation. The statistical index for the shoot proliferation coefficient was defined as the number of proliferating shoots divided by the number of explants.

### 2.3. Determining the Optimal Antibiotic Concentration for Adventitious Shoot Proliferation

From the 9th subculture, adventitious shoots were selected for experimentation 30 days post cultivation, with the length of the shoots approximately 3 cm and at least one leaf. WPM was used as the basal culture medium, supplemented with 30 g/L sucrose, 6 g/L agar, and 0.3 mg/L 6-benzylaminopurine (6-BA), with the pH adjusted to the same as above. Various concentrations of cefotaxime (Cef, BIORIGIN, BN24159) (0, 10, 50, 100, and 200 mg/L), Plant Preservative Mixture (PPM, 0.005, 0.01, 0.02, 0.05, and 0.1%), and timentin (Tim, BIORIGIN, BN24287) (Tim, 10, 50, 100, and 300 mg/L) were added to the medium (Supplemental Table S2). Fifteen shoots were prepared for each treatment twice, equivalent to thirty replicates. The number of days to achieve 80% shoot proliferation was recorded. The number of proliferated shoots was counted after 30 days of inoculation.

### 2.4. Determination of Optimal Phytohormone Concentration for Rooting of Adventitious Shoots

From the 8th subculture, adventitious shoots were selected for experimentation 30 days post cultivation, with the length of the shoots approximately 3 cm and at least one leaf. WPM was employed as the basal culture medium, supplemented with 30 g/L sucrose, 6 g/L agar, and 100 mg/L of the antibiotic cefotaxime, with the pH adjusted to the same as above. Various concentrations of 1-Naphthaleneacetic acid (NAA) (0.05, 0.1, 0.2, 0.5, and 1 mg/L) or Indolebutyric acid (IBA) (0.2, 0.4, 0.8, 1, and 1.6 mg/L) were added to the basal medium to facilitate the regeneration of seedlings (Supplemental Table S3). Each treatment was replicated 30 times, as above. After 30 days of inoculation, the root induction rate, time to root, average number of roots, average root length, and the longest root length were statistically analyzed.

### 2.5. Acquisition of Optimal Rooting Basal Medium

Different basal media, namely Murashige and Skoog (MS), half-strength MS (1/2MS), quarter-strength MS (1/4MS), and Woody Plant Medium (WPM), were utilized. Each medium was supplemented with 30 g/L sucrose, 6 g/L agar, 100 mg/L of the antibiotic cefotaxime, and 0.05 mg/L of 1-Naphthaleneacetic acid (NAA), with the pH adjusted to between 5.70 and 5.84. Each treatment was replicated 30 times, as above. From the 9th subculture, adventitious shoots were selected for experimentation, with the length of the shoots approximately 3 cm and at least one leaf. After 30 days of inoculation, the root induction rate, time to root, average number of roots, average root length, and the longest root length were statistically assessed.

### 2.6. Statistical Analysis

The data were organized using Excel 2021 and SPSS 29. The significance of differences among treatments was determined using one-way analysis of variance (ANOVA). The *p*-values were calculated using the Student’s t-test (*p* < 0.05).

## 3. Results

### 3.1. Effects of TDZ and 6-BA on Shoot Proliferation in Q. robur

**Figure 1:**
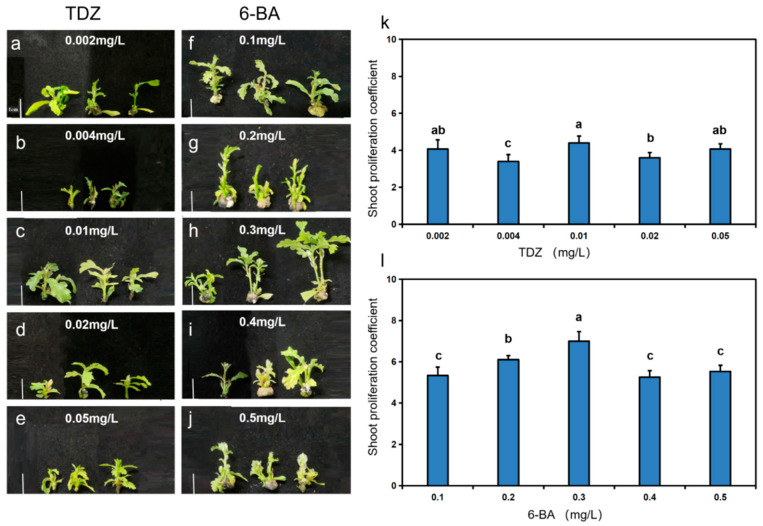
Phenotypes of *Q. robur* shoot proliferation under different hormone treatments. (**a**–**j**) Phenotypes of *Q. robur* shoot proliferation under TDZ (0.002, 0.004, 0.01, 0.02, and 0.05 mg/L) and 6-BA (0.1, 0.2, 0.3, 0.4, and 0.5 mg/L) treatments at 30 d, bar = 1 cm. (**k**) Effect of different concentrations of TDZ on the shoot proliferation coefficient of *Q. robur*. (**l**) Effect of different concentrations of 6-BA on the shoot proliferation coefficient of *Q. robur*. Letters on each bar indicate significant differences, *p* < 0.05. Analyses were performed using a one-way ANOVA test and data are means ± standard error (*n* = 30).

To explore the effects of TDZ and 6-BA on shoot proliferation, healthy shoots of *Q. robur* with consistent growth were selected for tissue culture. The number of days to achieve 80% shoot proliferation varied from 5 to 13 days across different concentrations of TDZ, with shoot proliferation coefficients significantly differing, ranging from 3.4 to 4.4 (Supplemental Table S4, Figure 1a–e,k). The most rapid proliferation and the highest coefficient were observed at a TDZ concentration of 0.01 mg/L (Supplemental Table S4). The ANOVA results indicated a significant difference (*p* < 0.05) in the proliferation effect of *Q. robur* shoots at 0.004 mg/L, 0.01 mg/L, and 0.02 mg/L TDZ concentrations (Supplemental Table S4).

Similarly, the number of days to reach 80% shoot proliferation with varying concentrations of 6-BA ranged from 5 to 8 days, with shoot proliferation coefficients significantly differing, ranging from 5.25 to 6.92 (Supplemental Table S4, Figure 1f–j,l). The fastest proliferation rate and the highest coefficient were noted at a 6-BA concentration of 0.3 mg/L (Supplemental Table S4). Analysis revealed a significant difference (*p* < 0.05) between 0.3 mg/L and 0.2 mg/L 6-BA concentrations and the other concentrations tested (Supplemental Table S4).

### 3.2. Effects of Different Antibiotics on Shoot Proliferation in Q. robur

**Figure 2:**
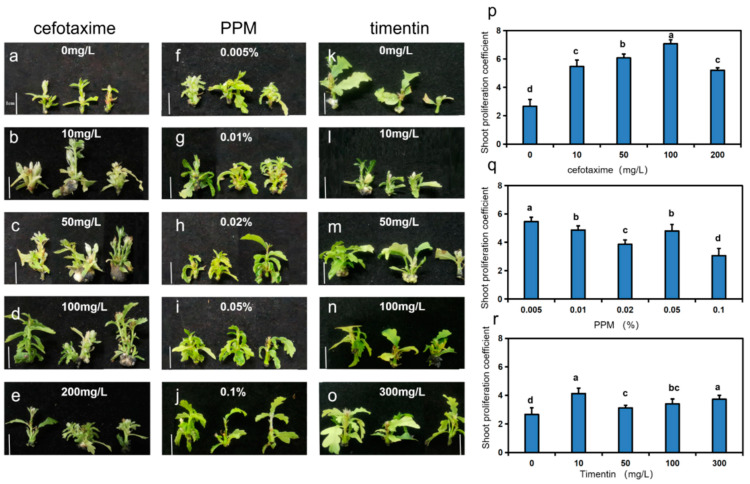
Phenotypes of *Q. robur* shoot proliferation under different antibiotic treatments. (**a**–**o**) Phenotypes of *Q. robur* shoot proliferation under different antibiotic treatments at 30 d, bar = 1 cm. (**p**) Effect of different concentrations of cefotaxime on the shoot proliferation coefficient of *Q. robur*. (**q**) Effect of different concentrations of PPM on the shoot proliferation coefficient of *Q. robur*. (**r**) Effect of different concentrations of timentin on the shoot proliferation coefficient of *Q. robur*. Letters on each bar indicate significant differences, *p* < 0.05. Analyses were performed using a one-way ANOVA test and data are means ± standard error (*n* = 30).

To explore the effects of different antibiotics on shoot proliferation in *Q. robur*, we recorded and compared the number of days needed to achieve 80% shoot proliferation, which ranged from 6 to 10 days at various cefotaxime concentrations, along with significantly differing shoot proliferation coefficients, ranging from 2.67 to 7.07 (Supplemental Table S5, Figure 2a–e,p). The quickest proliferation time and the highest coefficient—6 days and 7.07, respectively—were both observed at a cefotaxime concentration of 100 mg/L, which was significantly higher than the other groups (Supplemental Table S5).

The time to reach 80% shoot proliferation at different PPM concentrations ranged from 4 to 6 days, with coefficients of shoot proliferation significantly differing, from 3.07 to 5.47 (Supplemental Table S5, Figure 2f–j,q). The fastest proliferation time and the highest coefficient of 5.47 were found at a PPM concentration of 0.005%, significantly higher than the other groups (Supplemental Table S5). The ANOVA results demonstrated a significant difference (*p* < 0.05) in the effect of PPM on the shoot proliferation of *Q. robur* (Supplemental Table S5), with 0.01% and 0.005% PPM reaching 80% shoot proliferation the quickest (Supplemental Table S5). The optimal concentration of PPM for inducing shoot proliferation in *Q. robur* was thus concluded to be 0.005%.

Furthermore, the time to reach 80% shoot proliferation at various timentin concentrations was 6–7 days, with shoot proliferation coefficients significantly differing, from 2.67 to 4.13 (Supplemental Table S5, Figure 2k–o,r). The quickest proliferation time was observed at 100 mg/L, while the highest coefficient of 4.13 was at 10 mg/L (Supplemental Table S5). The results indicated that the highest shoot proliferation coefficient occurred at a timentin concentration of 10 mg/L, with no significant difference between the 300 mg/L and 100 mg/L groups, but both were significantly greater than the other groups (Supplemental Table S5).

### 3.3. Effects of NAA and IBA on Rooting Rate and Root Growth in Q. robur

**Figure 3:**
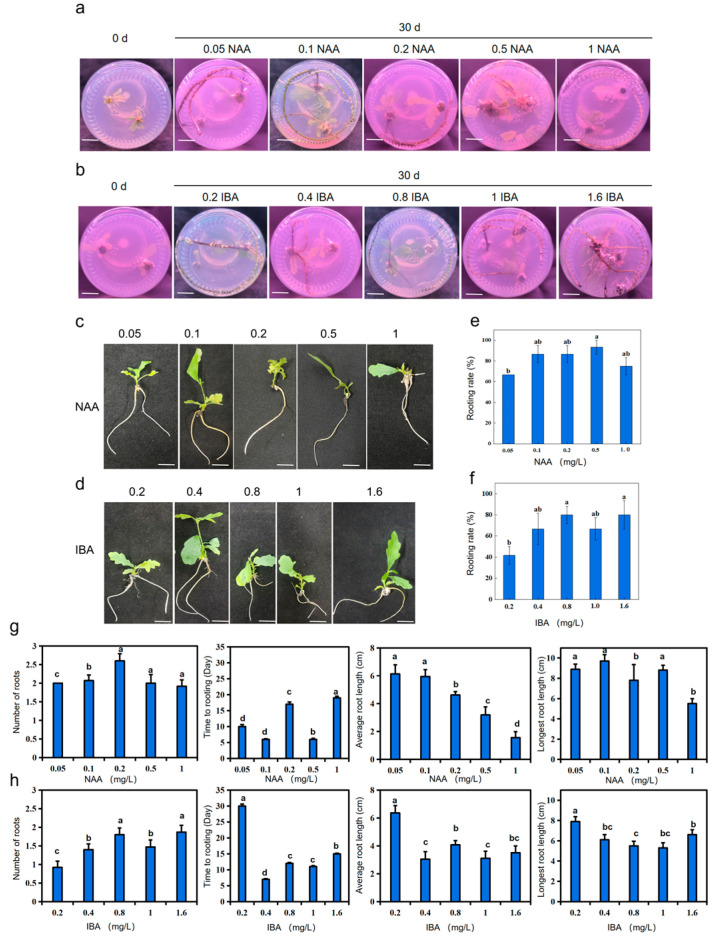
Phenotypes of adventitious root formation of *Q. robur* in media containing different concentrations of NAA and IBA. (**a**,**b**) Phenotypes of *Q. robur* rooting under different concentrations of NAA and IBA treatment at 30 d, bar = 1 cm. (**c**,**d**) Individual seedling phenotypes of adventitious root formation in *Q. robur* under different concentrations of NAA and IBA treatment at 30 d, bar = 1 cm. (**e**,**f**) Effect of different concentrations of NAA and IBA on rooting rate of *Q. robur*. Letters on each bar indicate significant differences, *p* < 0.05. Data are means ± standard error (*n* = 30). (**g**,**h**) Analysis of rooting time, average number of roots, average root length, and longest root length of inoculated seedlings under different concentrations of NAA and IBA. Letters on each bar indicate significant differences, *p* < 0.05. Data are means ± standard error (*n* = 30).

A series of experiments were conducted to explore the effects of NAA and IBA rooting rate and root growth in *Q*. *robur*. In the NAA group, a concentration of 0.5 mg/L yielded the highest rooting rate of 93.33%, significantly higher than the other groups (Supplemental Table S6, Figure 3e). Low concentrations of NAA resulted in an inconspicuous rooting rate, while high concentrations were detrimental to adventitious root formation in *Q. robur* (Supplemental Table S6, Figure 3a,c,e,g). Post inoculation, the number of adventitious roots, root length, and other indices were recorded and photographed (Figure 3a,c,e,g). At 30 days, the average number of rooted strips was highest at 0.1 mg/L, with an average of 2.07, significantly higher than the other concentrations tested (Figure 3g). The number of rooted strips at 0.05 mg/L was 2.00, indicating that low NAA concentrations did not significantly promote the average number of rooted strips, while high concentrations were also detrimental to root proliferation in *Q. robur* (Figure 3g). This trend mirrored the rooting rate under the corresponding NAA concentrations. The shortest time to achieve 80% rooting was 6 days at 0.1 mg/L and 0.5 mg/L, significantly lower than the other concentrations (Supplemental Table S6, Figure 3g). The longest root length at 30 days was 8.9 cm at 0.1 mg/L, significantly longer than the groups at 0.2 mg/L and 1 mg/L concentrations. The difference among the other groups was not significant (Supplemental Table S6, Figure 3g). At 0.05 mg/L, the longest root length was 8.1 cm, only 0.8 cm shorter than at 0.1 mg/L, suggesting that lower NAA concentrations favor root elongation in *Q. robur* (Figure 3g). The longest average root length at 30 days was at 0.05 mg/L, with an average of 6.13 cm (Supplemental Table S6, Figure 3g). As the concentration increased, the average root length decreased, indicating that higher NAA concentrations result in shorter average root lengths (Supplemental Table S6, Figure 3g). Overall, 0.1 mg/L NAA was deemed optimal for adventitious root formation in *Q. robur*.

In the IBA group, the highest rooting rate of 80.00% was observed at concentrations of 0.8 mg/L and 1.6 mg/L, significantly higher than that at 0.2 mg/L (Supplemental Table S6, Figure 3b,d,f,h). No significant difference was found between the rooting rates of 0.4 mg/L and 1.0 mg/L IBA groups, with both at 67.00% (Supplemental Table S6, Figure 3f). Post inoculation, the number of roots, root length, and other indices were regularly recorded and photographed (Figure 3h). At 30 days, the longest average root length was at 0.2 mg/L, with an average of 6.36 cm, significantly higher than the other concentrations (Supplemental Table S6, Figure 3h). The remaining groups had average lengths of 3.56 cm, 4.07 cm, 3.05 cm, and 3.11 cm, indicating that high IBA concentrations were not conducive to *Q. robur* root elongation (Figure 3h). The longest root length at 30 days under 0.2 mg/L was 7.9 cm, significantly longer than the other groups (Supplemental Table S6, Figure 3h). The longest root lengths of the other groups were less than 6 cm, suggesting that higher IBA concentrations hinder root elongation in *Q. robur* (Figure 3h). The highest average number of rooted strips at 30 days was at 1.6 mg/L, with an average of 1.87, which is significantly higher than the other concentrations tested (Supplemental Table S6, Figure 3h). The difference in the 0.8 mg/L group was not significant, indicating that low IBA concentrations had little effect on the average number of rooted strips, while high concentrations promoted more adventitious root formation, similar to the rooting rate trend under the corresponding IBA concentrations. The shortest average rooting time was 7 days at 0.4 mg/L, which is significantly lower than the other concentrations (Supplemental Table S6, Figure 3h).

### 3.4. Effect of Different Basal Media on Rooting Rate and Root Growth in Q. robur

**Figure 4:**
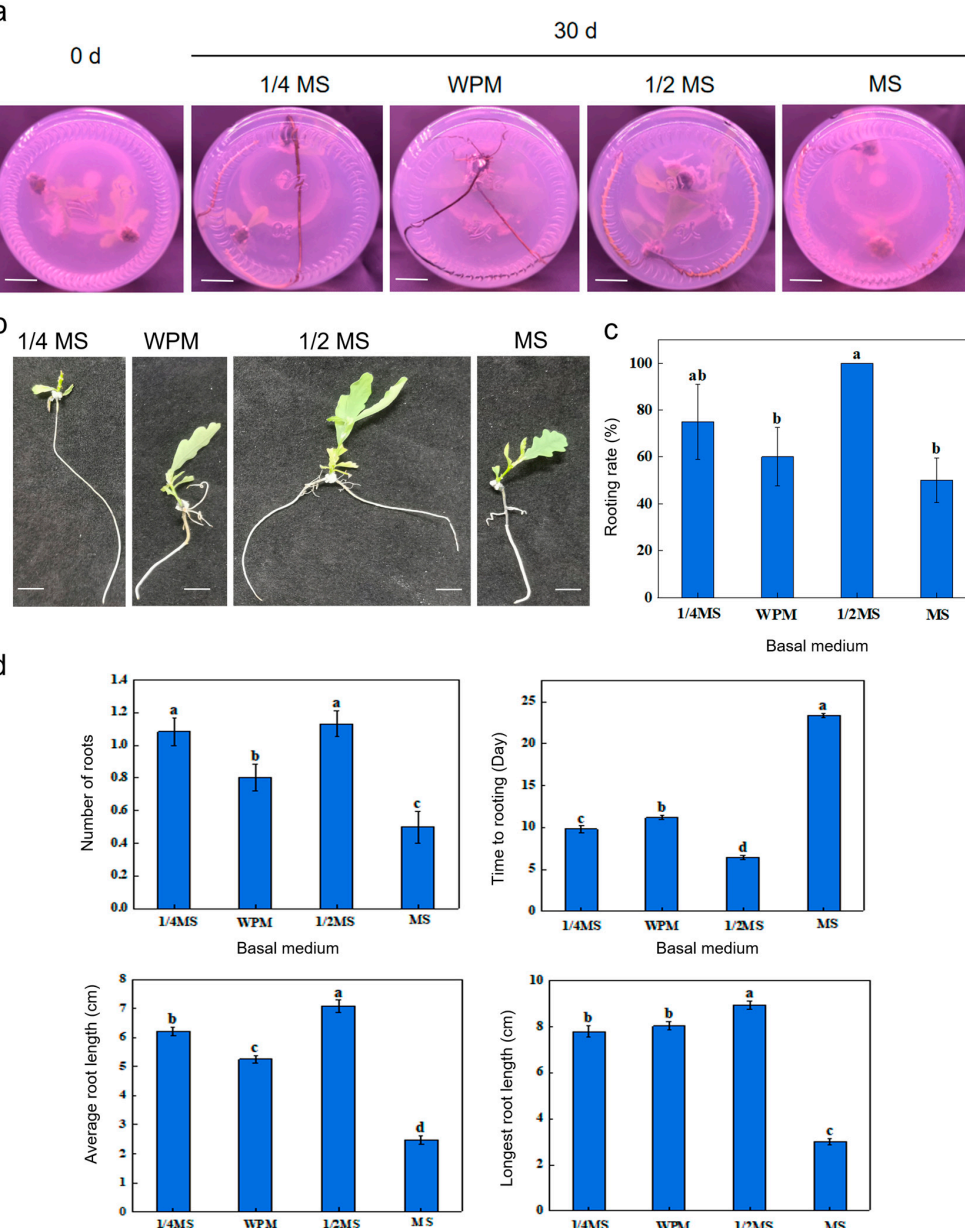
Phenotypes of adventitious root formation of *Q. robur* in media containing different basal media. (**a**) Phenotypes of *Q. robur* rooting under different basal media at 30 d, bar = 1 cm. (**b**) Individual seedling phenotypes of adventitious root formation in *Q. robur* under different basal media at 30 d, bar = 1 cm. (**c**) Effect of different basal media on rooting rate of *Q. robur*. Letters on each bar indicate significant differences, *p* < 0.05. Data are means ± standard error (*n* = 30). (**d**) Analysis of rooting time, average number of roots, average root length, and longest root length of inoculated seedlings under different basal media. Letters on each bar indicate significant differences, *p* < 0.05. Data are means ± standard error (*n* = 30).

To explore the effect of different basal media on rooting rate and root growth in *Q. robur*, experiments were conducted on selected shoots of *Q. robur* with growth conditions consistent with other groups. The highest rooting rate of 100% was achieved in the 1/2MS medium, which is significantly higher than the WPM and MS media, with no significant difference from 1/4MS (Supplemental Table S7, Figure 4c). The 1/4MS group achieved 75% rooting, WPM 60%, and MS 50% (Supplemental Table S7, Figure 4c).

By recording the number of roots, root length, and other indices, we found that at 30 days, the longest average root length was in 1/2MS, with an average of 7.08 cm, which is significantly higher than in WPM and MS and not significantly different from 1/4MS (Supplemental Table S7, Figure 4a,b,d). The average numbers of rooting strips for 1/4MS, WPM, and MS were 6.20 cm, 5.25 cm, and 2.48 cm, respectively (Supplemental Table S7, Figure 4d), indicating that 1/2MS favored root elongation in *Q. robur*, similar to the rooting rate trend under the corresponding medium compositions.

The longest root length at 30 days was in 1/2MS, with the longest root being 9.4 cm, significantly longer than the WPM, MS, and 1/4MS groups (Supplemental Table S7, Figure 4d). The average numbers of rooting strips for 1/4MS, WPM, and MS were 8.5 cm, 8.7 cm, and 5.0 cm, respectively, indicating that 1/2MS favored root elongation in *Q. robur*, similar to the rooting rate trend under the corresponding medium compositions. At 30 days, the highest average number of rooting strips was in the 1/2MS group, with an average of 1.132, which is significantly higher than the WPM, MS, and 1/4MS groups (Supplemental Table S7, Figure 4d). The average numbers of rooting strips were 1.083, 0.802, and 0.500 for 1/4MS, WPM, and MS, respectively, indicating that 1/2MS more favored rooting in *Q. robur* (Supplemental Table S7, Figure 4d), similar to the rooting rate trend under the corresponding medium compositions. The shortest average rooting time at 30 days was in the 1/2MS group, which was 7 days (Supplemental Table S7, Figure 4d). It was significantly lower than the mean rooting time of the 1/4MS, WPM, and MS groups.

## 4. Discussion

The direct organogenesis regeneration method, as opposed to indirect regeneration through callus formation, significantly reduces the time required for obtaining regenerated plants, a finding that aligns with recent studies [26]. Although the literature is replete with research on shoot proliferation and adventitious root formation in *Q. robur*, such as Chalupa’s work [25], these studies are now considered outdated and their applicability to the current production practices is limited. This underscores the urgent need for further exploration of more effective induction conditions.

The role of plant exogenous hormones in growth, development, and metabolic regulation is well established, with varying effects observed depending on the type and concentration of plant hormones used [27,28]. However, there is a dearth of research on the specific roles of individual hormones, different antibiotics, and optimal culture media. These factors are crucial for shoot proliferation and adventitious root formation, processes that directly impact seedling survival and growth post transplantation. According to Vengadesan et al., the use of Woody Plant Medium (WPM) as a basal medium, supplemented with cytokinins and growth factors, has been shown to enhance somatic embryogenesis and improve root development outcomes in *Q. rubra* [29].

For shoot proliferation cultures, 0.3 mg/L 6-BA was identified as the most suitable phytohormone and concentration, surpassing 0.01 mg/L TDZ in terms of the shoot proliferation coefficient (Supplemental Table S4). In terms of antibiotic selection, 100 mg/L cefotaxime was the optimal choice, yielding the highest shoot proliferation coefficient and a shoot proliferation time only two days longer than the fastest group (Supplemental Table S5). For adventitious root formation culture, a combination of indicators suggests that 0.1 mg/L NAA is the most suitable hormone and concentration, offering a more advantageous and cost-effective solution, despite some individual indicators not surpassing 0.5 mg/L NAA (Supplemental Table S6). For adventitious root formation culture and for the choice of base medium, the 1/2 MS medium was the most suitable, demonstrating clear advantages across all evaluated aspects (Supplemental Table S7).

In the study conducted by Chalupa et al., the optimal formulation for the proliferation of *Q. robur* shoots and the formation of adventitious roots was explored. Their research indicated that adding 0.6 mg/L PBA to the WPM yielded the best results for shoot multiplication, with the average increase in the number of multiplied shoots reaching 3.1 [25]. However, in our study, the incorporation of 0.3 mg/L 6-BA and 100 mg/L cefotaxime into the WPM achieved a multiplication efficiency of 7.07, which significantly outperforms the shoot multiplication medium identified by Chalupa et al. Furthermore, Chalupa et al. discovered that the addition of 1 mg/L Indolebutyric acid (IBA) to the 1/2 WPM induced rooting in adventitious shoots within approximately two weeks, with a rooting rate of 84% [25]. Our findings revealed that the addition of 0.05 mg/L NAA to the 1/2 MS medium resulted in 80% of the adventitious shoots initiating rooting within just seven days, with a rooting rate of 100%. Compared to the rooting medium formulation obtained by Chalupa et al., the formulation achieved in this study not only reduced the time required for adventitious roots to initiate rooting, but also significantly increased the rooting rate. The present study has successfully developed a protocol for the multiplication and rooting of *Q. robur* adventitious shoots, enabling the rapid acquisition of a large number of sterile seedlings. Previous studies have established WPM as the most suitable medium for the tissue culture of woody plants [30], but our investigation into the optimal medium revealed that the 1/2MS medium yielded the best rooting outcomes when the concentration of NAA was at precisely 0.05 mg/L. There are many ancient trees extant within *Q. robur*, and our study may also have some significance for the conservation of ancient trees, especially if the seeds of ancient trees can be collected, which may make it easier to obtain excellent germplasm resources than it is by using the sterilized exuviae method [15]. This advancement plays a crucial role in promoting superior varieties of *Q. robur*, enhancing the ecological benefits of this species, and providing a foundation for optimized tissue culture techniques that supports efforts to conserve plant genetic resources and breeding.

However, this study has its limitations. Firstly, the experiment explored a limited concentration range of plant hormones, such as 6-BA, TDZ, NAA, IBA, and antibiotics like cefotaxime, PPM, and timentin, indicating a need for further exploration of hormone and antibiotic combinations in future studies. Secondly, orthogonal experiments to select the most appropriate formulation of hormones, antibiotics, and basal media were not conducted. This finding necessitates further research to elucidate the most appropriate medium conditions for rooting in *Q. robur*. Additionally, previous research has indicated that temperature influences shoot proliferation and light affects rooting in *Q. robur* [31,32]. The RITA^®^ Bioreactor (VITROPIC, Saint-Mathieu-de-Tréviers, France) is well worth trying for shoot proliferation, and although it does not have much of a beneficial effect in ancient *Q. robur*, it may be able to have a significant impact on seedling shoot proliferation [15]. Consequently, we intend to integrate these factors into our subsequent studies, with the goal of achieving the optimal conditions for the tissue culture of *Q. robur*.

## 5. Conclusions

The experimental data and statistical analysis have led to the conclusion that the addition of 0.3 mg/L 6-BA and 100 mg/L cefotaxime to WPM significantly enhances shoot proliferation in *Q. robur*. This experiment improved the shoot proliferation coefficient by approximately 0.5 to 2.5 times compared to the ratios and concentrations proposed in previous studies. By investigating the effects of individual hormones and selecting the best medium, we achieved an 86.67% rooting rate in the 0.1 mg/L NAA group, with an average number of rooting strips of about 2.07 and an average root length of about 5.95 cm. The use of the 1/2 MS medium resulted in a 100.00% rooting rate, with an average number of rooting strips of about 1.13, and an average root length of about 7.08 cm. By combining the optimal conditions, except for basal media, we have developed an efficient method using WPM for the tissue culture regeneration of *Q. robur*, as shown in Supplemental Figure S1. This method offers robust technical support for the rapid propagation and environmental adaptation studies of *Q. robur*.

## Data Availability

Data will be made available on request.

## Supplementary Materials

The following supporting information can be downloaded at: https://www.mdpi.com/article/10.3390/life15030348/s1, Table S1: Experimental design of shoot proliferation experiments with different concentrations of TDZ and 6-BA. Table S2: Experimental design of shoot proliferation experiments with different concentrations of cefotaxime, PPM, and timentin. Table S3: Experimental design for adventitious root formation with different concentrations of NAA and IBA. Table S4: Effects of different concentrations of TDZ and 6-BA on the shoot proliferation of *Q. robur*. Table S5: Effects of different concentrations of cefotaxime, PPM, and timentin on the shoot proliferation of *Q. robur*. Table S6: Effects of different concentrations of NAA and IBA on the adventitious root formation of *Q. robur*. Table S7: Effects of different basal media on the adventitious root formation of *Q. robur*. Figure S1: The process of rooting and shoot proliferation of *Q. robur* from seeds in the best conditions combination.
