# Supplementary material for: Exploration of Suitable Conditions for Shoot Proliferation and Rooting of Quercus robur L. in Plant Tissue Culture Technology
Source: Life (Basel). 2025 Feb 23;15(3):348. doi: 10.3390/life15030348 (PMC11943399; doi:10.3390/life15030348)
Supplement: Supplementary file 1 [file life-15-00348-s001.zip › life-3427870-supplementary.pdf]

Supplemental Table S1. Experimental design of shoot proliferation experiments with different concentrations of TDZ and 6-BA

	Concentration (mg/L)	Concentration (mg/L)
TDZ	0.002	-
	0.004	-
	0.01	-
	0.02	-
	0.05	-
6-BA	-	0.1
	-	0.2
	-	0.3
	-	0.4
	-	0.5

Supplemental Table S2. Experimental design of shoot proliferation experiments with different concentrations of cefotaxime, PPM and timentin

	Concentration (mg/L)	Concentration (%)	Concentration (mg/L)
CK	0	0	0
cefotaxime	10	-	-
	50	-	-
	100	-	-
	200	-	-
PPM	-	0.005	-
	-	0.01	-
	-	0.02	-
	-	0.05	-
	-	0.1	-
timentin	-	-	10
	-	-	50
	-	-	100
	-		300

Supplemental Table S3. Experimental design for adventitious root formation with different concentrations of NAA and IBA

	Concentration (mg/L)	Concentration (mg/L)
NAA	0.05	-
	0.1	-
	0.2	-
	0.5	-
	1.0	-
IBA	-	0.2
	-	0.4
	-	0.8
	-	1.0
	-	1.6

Supplemental Table S4. Effects of different concentrations of TDZ and 6-BA on the shoot proliferation of *Q. robur*

Phytohormone	Concentration (mg/L)	Number of days to achieve 80% shoot proliferation (d)	Shoot proliferation coefficient
TDZ	0.002	13	4.07 ± 0.49^{ab}
	0.004	13	3.40 ± 0.37^{c}
	0.01	5	4.40 ± 0.37^{a}
	0.02	13	3.60 ± 0.28^{b}
	0.05	8	4.07 ± 0.28^{ab}
6-BA	0.1	8	5.33 ± 0.41^{c}
	0.2	6	6.11 ± 0.19^{b}
	0.3	5	7.00 ± 0.47^{a}
	0.4	5	5.25 ± 0.32^{c}
	0.5	8	5.53 ± 0.30^{c}

Supplemental Table S5. Effects of different concentrations of cefotaxime, PPM and timentin on the shoot proliferation of *Q. robur*

Antibiotics	Concentration (mg/L)	Number of days to achieve 80% shoot proliferation (d)	Shoot proliferation coefficient
cefotaxime	0	10	2.67 ± 0.47^{d}
	10	8	5.47 ± 0.45^{c}
	50	7	6.07 ± 0.28^{b}
	100	6	7.07 ± 0.28^{a}
	200	8	5.20 ± 0.18^{c}
PPM	0.005（%）	4	5.47 ± 0.30^{a}
	0.01（%）	4	4.87 ± 0.30^{b}
	0.02（%）	8	3.87 ± 0.30^{c}
	0.05（%）	5	4.80 ± 0.45^{b}
	0.1（%）	6	3.07 ± 0.49^{d}
timentin	0	7	2.67 ± 0.47^{d}
	10	7	4.13 ± 0.38^{a}
	50	7	3.13 ± 0.18^{c}

	100	6	3.42±0.32^{bc}
	300	7	3.73±0.28^{ab}

Supplemental Table S6. Effects of different concentrations of NAA and IBA on the adventitious root formation of *Q. robur*

Phytohormone	Concentration (mg/L)	Rooting rate (%)	Average number of roots	Days to achieve 80% rooting (d)	Length of the longest root (cm)	Average root length (cm)
NAA	0.05	66.67±0.00^{b}	2.00±0.00^{c}	10	8.9±0.50^{a}	6.13±0.65^{a}
	0.1	86.67±18.26^{ab}	2.07±0.15^{b}	6	9.7±0.64^{a}	5.95±0.48^{a}
	0.2	86.67±18.26^{ab}	2.60±0.19^{a}	17	7.8±1.55^{b}	4.62±0.53^{b}
	0.5	93.33±16.91^{a}	2.00±0.23^{a}	6	8.8±0.48^{a}	3.19±0.58^{c}
	1.0	75.00±16.67^{ab}	1.92±0.17^{a}	19	5.5±0.50^{b}	1.56±0.43^{d}
IBA	0.2	41.67±16.67^{b}	0.92±0.17^{c}	30	7.9±0.47^{a}	6.36±0.52^{a}
	0.4	66.67±33.34^{ab}	1.40±0.15^{b}	7	6.1±0.50^{bc}	3.05±0.53^{c}
	0.8	80.00±18.26^{a}	1.80±0.18^{a}	12	5.5±0.46^{c}	4.07±0.31^{b}
	1.0	66.87±	1.47±	11	5.3±	3.11±

		23.58^{ab}	0.19^{b}		0.50^{bv}	0.50^{c}
	1.6	80.00±29.82^{a}	1.87±0.18^{a}	15	6.6±0.48^{b}	3.50±0.50^{bc}

Supplemental Table S7. Effects of different basal medium on the adventitious root formation of *Q. robur*

Culture medium	Rooting rate (%)	Average number of roots	Days to achieve 80% rooting (d)	Length of the longest root (cm)	Average root length (cm)
1/4MS	75.00±31.92^{ab}	6.20±0.17^{a}	10	8.5±0.48^{b}	1.08±0.30^{b}
WPM	60.00±27.89^{b}	5.25±0.18^{b}	12	8.7±0.43^{b}	0.80±0.31^{c}
1/2MS	100.00±0.00^{a}	7.08±0.18^{a}	7	9.4±0.37^{a}	1.13±0.45^{a}
MS	50.00±19.25^{b}	2.48±0.20^{c}	23	5.0±0.29^{c}	0.50±0.26^{d}

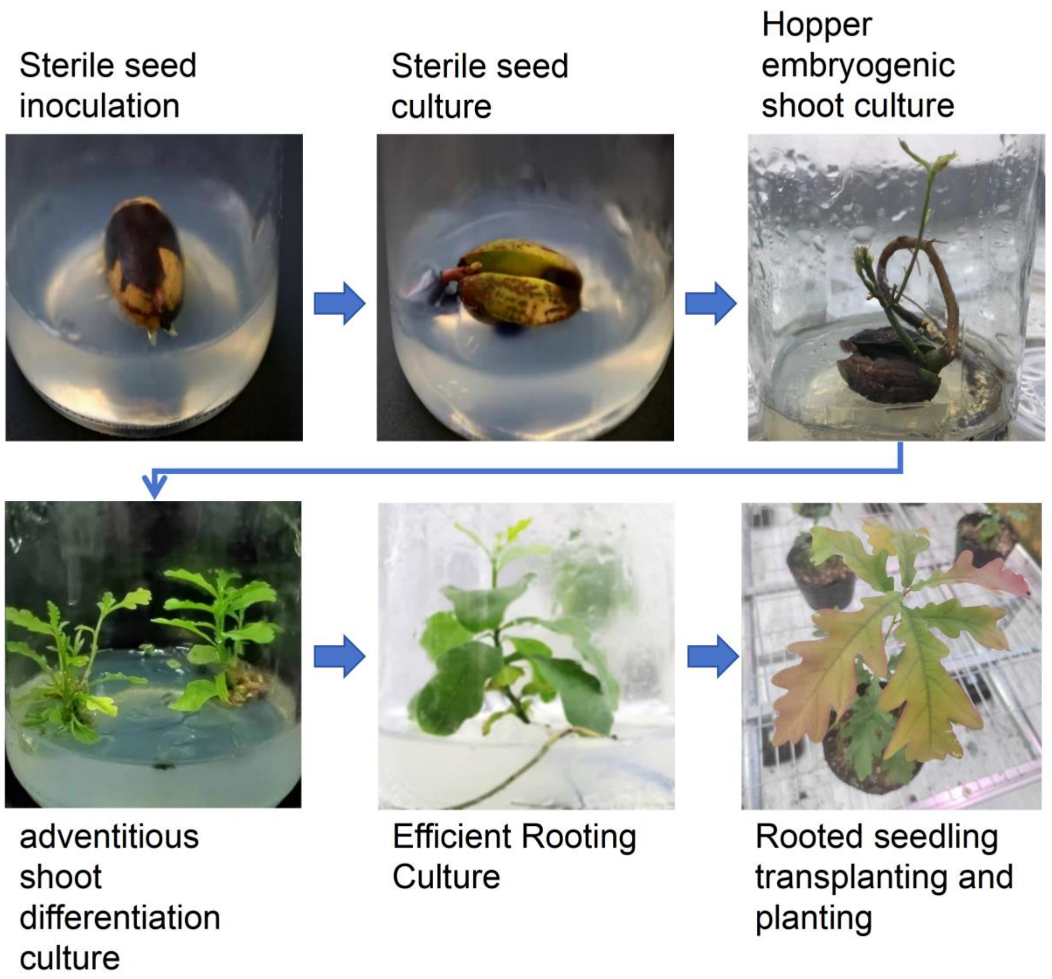

Supplemental Figure S1. The process of rooting and germination of *Q. robur* from seed in the best conditions combination.
